# Altered Regional Homogeneity in Pediatric Bipolar Disorder during Manic State: A Resting-State fMRI Study

**DOI:** 10.1371/journal.pone.0057978

**Published:** 2013-03-06

**Authors:** Qian Xiao, Yuan Zhong, Dali Lu, Weijia Gao, Qing Jiao, Guangming Lu, Linyan Su

**Affiliations:** 1 Key Laboratory of Psychiatry and Mental Health of Hunan Province, Mental Health Institute of The Second Xiangya Hospital, Central South University, Changsha, Hunan, China; 2 Department of Medical Imaging, Jinling Hospital, Nanjing University School of Medicine, Nanjing, Jiangsu, China; 3 School of Psychology, Nanjing Normal University, Nanjing, Jiangsu, China; Hangzhou Normal University, China

## Abstract

Pediatric bipolar disorder (PBD) is a severely debilitating illness, which is characterized by episodes of mania and depression separated by periods of remission. Previous fMRI studies investigating PBD were mainly task-related. However, little is known about the abnormalities in PBD, especially during resting state. Resting state brain activity measured by fMRI might help to explore neurobiological biomarkers of the disorder. Methods: Regional homogeneity (ReHo) was examined with resting-state fMRI (RS-fMRI) on 15 patients with PBD in manic state, with 15 age-and sex-matched healthy youth subjects as controls. Results: Compared with the healthy controls, the patients with PBD showed altered ReHo in the cortical and subcortical structures. The ReHo measurement of the PBD group was negatively correlated with the score of Young Mania Rating Scale (YMRS) in the superior frontal gyrus. Positive correlations between the ReHo measurement and the score of YMRS were found in the hippocampus and the anterior cingulate cortex in the PBD group. Conclusions: Altered regional brain activity is present in patients with PBD during manic state. This study presents new evidence for abnormal ventral-affective and dorsal-cognitive circuits in PBD during resting state and may add fresh insights into the pathophysiological mechanisms underlying PBD.

## Introduction

Pediatric bipolar disorder (PBD) is a severely debilitating illness, which is characterized by episodes of mania and depression separated by periods of remission. Nearly 1% of adolescents and children have diagnosis of PBD [1]. One study showed that nearly 65% of PBD attempted suicide and almost 75% had been in hospital [2]. Compared to adults, a juvenile onset PBD leads to have a higher rate of suicide and substance abuse behavior [3]. Currently, diagnosis of this disorder is based almost entirely on a review of clinical history. The complaints of patients themselves play an important part for diagnosis. Young people will not describe their symptoms readily or easily, even to their parents. Thus, neurobiological studies on pediatric BD may help illustrate the pathophysiological mechanisms of PBD.

So far, functional studies investigating PBD have mainly used emotional or cognitive process tasks. Using varied kinds of emotional tasks,findings in PBD exhibited either reduced or increased activity in the ventrolateral prefrontal cortex (VLPFC) [4], [5], [6], [7] dorsolateral prefrontal cortex (DLPFC) [4], [5], [8] and anterior cingulate cortex (ACC) [9], [10] relative to healthy controls (HC). Moreover, the PBD subjects had significantly reduced connectivity between the left amygdala and the right posterior cingulate cortex and the right parahippocampal gyrus (PHG) in the face emotion processing, relative to HC [11].In the cognitive tasks, PBD showed increased activity in the DLPFC [8], [12], superior frontal cortex [13], putamen and thalamus [14]. Pavuluri et al. probed emotion and cognition processes simultaneously employing emotional Stroop task in PBD. PBD patients showed increased activity in the ACC and the amygdala and reduced activity in the VLPFC and DLPFC [15].

Many studies have proposed structural alterations in the cortical [16] and subcortical [8], [14], [17] areas. Studies in children and adolescent bipolar disorder [16], [17], [18], [19], [20], [21], [22] and one longitudinal study of PBD [17] showed smaller amygdala, which differed from the previous findings of enlarged amygdala in adults. While examining prefrontal volumes, PBD exhibited decreased volume of DLPFC [16] and VLPFC [23]. Other recent findings demonstrated decreased volume of superior temporal gyrus (STG), hippocampus, striatum and ACC in adolescents with bipolar disorder [16], [20], [24], [25].

To fully understand the neural pathophysiology of PBD, we need to investigate how the brain allocates most of its resources. However, task-dependent altered neural activity only represents no more than 5% of the total brain activity [26], [27]. Resting-state fMRI (RS-fMRI) is task-independent and can reflect endogenous neurophysiological process and spontaneous neuronal synchronization of the brain [27], [28]. There has been only one study in PBD analyzed by functional connectivity using RS-fMRI, which found negative resting state functional connectivity (RSFC) between the DLPFC and the STG [29]. RSFC used the correlation coefficients of all brain areas with a given region of interest (ROI). It is uncertain which brain area is abnormal when one area shows abnormal functional connectivity with other areas. Thus, it is important to measure the regional activity.

Regional homogeneity (ReHo), a newly developed method, reflects the temporal homogeneity of the regional blood oxygen level-dependent (BOLD) signal rather than its density, and serves as a complement for RSFC. Abnormal ReHo may be relevant to the changes of temporal aspects of neural activity in the regional area, and can be used to find abnormal activity in the whole brain [30]. The method does not demand a preliminary hypothesis such as a defined volume of interest for correlation analysis. Besides the functional connectivity analysis, the ReHo method can provide new insights in analyzing disease-related RS-fMRI data. To our knowledge, there have been no RS-fMRI studies on PBD patients using the ReHo method.

According to recent reviews, Pavuluri and Wessa [31], [32] described that bipolar disorder (BD) and PBD showed a dysfunctional ventral-affective brain network, encompassing the VLPFC, the ventral/subgenual anterior cingulate cortex (vACC), the amygdala, the insula, the striatum, and a hypoactive dorsal-cognitive network, including the DLPFC, the VLPFC, the dorsal anterior cingulate cortex (dACC), the posterior cingulate cortex (PCC) and the striatum. These ventral and dorsal networks are responsible for emotional and cognitive regulation, respectively.

We hypothesized that abnormal activity in the ventral-affective and dorsal-cognitive neural systems could be found in PBD. Our aim was to find out whether the abnormal regional activity detected by RS-fMRI and analyzed by the ReHo method in PBD patients differed from that in healthy controls.

## Materials and Methods

### Subjects

Twelve to seventeen-year old subjects were enrolled at Mental Health Institute of The Second Xiangya Hospital, Key Laboratory of Psychiatry and Mental Health of Human Province, Central South University (Changsha, P. R. China) and were scanned as soon as possible after initial contact (within 1 to 2 days). Healthy comparison participants (HC) were recruited through local middle school. The study was approved by the ethical committee of the Second Xiangya Hospital, and written informed consents were obtained from both the children and their parents. Recruitment included advertisements in physician offices and advertisements in local middle school.

The PBD patients (n = 15) were outpatients from psychiatric clinic in the Second Xiangya Hospital. The inclusion criteria were: meeting DSM-IV-TR criteria for BD, including at least one episode meeting full DSM-IV-TR criteria for hypomania (≥4 days) or mania (≥7 days),and being manic at the time of screening [31]. Exclusion criteria were: BD not otherwise specified, IQ≤80, autistic or Asperger’s disorder; schizophrenia; schizo-affective psychosis; medical illness that was unstable or could cause psychiatric symptoms; pregnancy; or substance abuse within ≤2 months of participation. In order to reduce comorbidity confounder in our results, we excluded PBD patients with comorbid attention deficit hyperactivity disorder (ADHD) or comorbid anxiety disorder.

For healthy controls (n = 15), the inclusion criteria were a negative history of psychiatric illness in the subject and his/her first-degree relatives. Exclusion criteria were IQ≤80; ongoing medical or neurological illness; pregnancy; or past/present psychiatric or substance disorder.

All participants were evaluated by a board-certified child psychiatrist using the Affective Disorders and Schizophrenia Scale for School-Age Children-Present and Lifetime Version (K-SADS-PL) administered to parents and children separately [33]. Information from this interview and all other available clinical information were reviewed to make a consensus clinical diagnosis. Comorbid diagnosis for BD youths was assessed by inquiring about symptoms during a time of relative euthymia to ensure that the PBD symptoms were not double-counted (Table 1).

**Table 1:** Participant Demographic Data in Pediatric BD Versus Typically Developing HC.

Group	PBD (M±SD)	HC (M±SD)	P value
Age	15.0±1.7	14.1±1.5	0.15*
Full-Scale IQ	97.9±10.62	101.3±5.58	0.282*
Gender (Male/female)	6/9	6/9	1.0#
Race (yellow/other)	15/0	15/0	1.0#
Handness (left/right)	0/15	0/15	1.0#
YMRS	36.5±4.1		
MFQ	10.3±4.3		
Current Comorbid KSADS			
Diagnoses			
Obsessive-compulsive disorder	2 (13.3%)		
Psychosis	3 (20% )		
Medications: Number and %			
of BD Participants			
Lithium	6 (40%)		
Atypical antipsychotics	11 (73.3%)		
Antidepressants	2 (13.3%)		
Valproate	8 (53.3%)		
Benzodiazepines	1 (6%)		
Familial history	1 (6%)		

Pediatric bipolar disorder (BD) (n = 15) vs. healthy control subjects (HC) (n = 15).

KSADS, Affective Disorders and Schizophrenia for School-Age Children-Present and Lifetime Version.

YMRS, Young Mania Rating Scale.

MFQ, Mood and Feelings Questionnaire.

# Pearson χ2 two-tailed test.

* Independent Sample T Test.

All participants were completed the Wechsler Abbreviated Scale of intelligence as an overall measure of cognitive ability [34]. The PBD patients were also completed the evaluation of Young Mania Rating Scale (YMRS) [35] and the Mood and Feelings Questionnaire (MFQ) [36]. All the scales were completed on the day of scanning. According to the division method proposed by Rich and Wood et al [11], [36], the manic state score was set at YMRS>26, MFQ<18; hypomanic state at 26>YMRS>12, MFQ<18. Handedness was assessed using the Edinburgh Inventory [37].

Statistical analysis was performed with SPSS11.5 software. Continuous data and categorical data were analyzed with t-tests and chi-square ^2^tests, respectively. Correlation between the YMRS score and activity of brain areas was assessed using Pearson’s correlations analysis.

### fMRI Data Acquisitions

Functional images were obtained on a 3-Tesla scanner (Siemens Trio) using a standard whole-head coil. Functional images were acquired by using of single-shot gradient echo-echo imaging (GRE-EPI) sequence (repetition time: 2000 ms; echo time: 30 ms; slices: 30; thickness: 4 mm; gap: 0.4 mm; field of view: 240 mm×240 mm; in-plane resolution: 64×64; flip angle: 90°).

Structural images were acquired by using a three-dimensional magnetization-prepared rapid gradient-echo (MPRAGE) sequence (repetition time: 2300 ms; echo time: 2.98 ms; inversion time: 900 ms; field of view: 256 mm ×256 mm; flip angle: 9°; in-plane resolution: 256×256). During the scanning, all subjects were informed to relax, hold still, keep their eyes closed without falling asleep and think of nothing in particular.

### fMRI Data Preprocessing

Spatial preprocessing of fMRI data was done using the SPM8 software (http://www.fil.ion.ucl.ac.uk/spm). The fMRI images were corrected for the acquisition delay between slices and for the head motion. The participants whose head motion exceeded 1.5 mm or rotation exceeded 1.5° during fMRI scanning were excluded. The fMRI images were normalized to a standard SPM8 echoplanar imaging template, resampling to 3×3×3 mm^3^. Data were temporal band-pass filtered (0.01–0.08 Hz) to reduce the effects of low-frequency drift and physiological high-frequency noise, and the linear trend was removed [38].

### Regional Homogeneity Measurement

Regional homogeneity analysis was performed with software REST (http://www.resting-fmri.sourceforge.net). ReHo maps were generated for each subject by calculating Kendall coefficient of concordance (KCC) of the time series of a given voxel and the nearest voxels (26 voxels) in a voxelwise analysis [30], [38]. The KCC value was calculated to this voxel, and an individual KCC map was acquired for each participant. Then a mask (made from the MNI template to assure matching with the normalization step), in the REST software, was used to remove non-brain tissue and for standardization purposes. Each individual ReHo map was divided by its own global mean KCC value within the mask [38]. Finally, the data were smoothed with a Gaussian filter of 4 mm full width at half-maximum (FWHM) to reduce noise and residual differences in gyral anatomy.

### Group Analyses of ReHo

One-sided one-sample t test (P<0.001, with family-wise error (FWE) rate correction) was first performed for both groups to show where the standardize KCC value was larger than 1 [39]. Then, voxelwise two-sample t tests were applied to compare the results in KCC between PBD patients and controls. The t-map was set at a corrected significance level of P<0.05 (combined height threshold P<0.01 (T >2.46) and a minimum cluster size of 18 voxels). Threshold value was determined using Monte Carlo simulation (AlphaSim: individual voxel P value = 0.01, 1000 simulations; FWHM = 4 mm, with mask) [40].

Finally, to study whether the ReHo correlates with the manic severity in the patients with PBD, a correlation analysis of ReHo versus YMRS score was performed in the patients with PBD at regions of interest (regions showed different ReHo in the above between-group ReHo analysis). For correlation analysis, a threshold of P<0.05 was chosen as a threshold for statistical significance.

## Results

### Participants

The PBD and HC groups did not differ significantly in age, gender, full-scale IQ and handedness. The PBD sample consisted of 15 participants with type I BD; none had type II BD, although it was not excluded. Our PBD participants were manic by mood ratings (YMRS 36.5±4.1, MFQ 10.3±4.3). All PBD participants were receiving psychopharmacological treatments, including lithium (n = 6 [40%]), atypical antipsychotics (n = 11 [73.3%]), Valproate (n = 8[53.3%]), antidepressants (n = 2 [13.3%]), and benzodiazepines (n = 1[6%]). Two PBD participants had comorbid obsessive-compulsive disorder (OCD) and three PBD participants had psychiatric symptoms. Only one PBD participants (6%) had one first-degree relative with BD. (Table 1).

### Between-group ReHo Analysis

**Figure 1:**
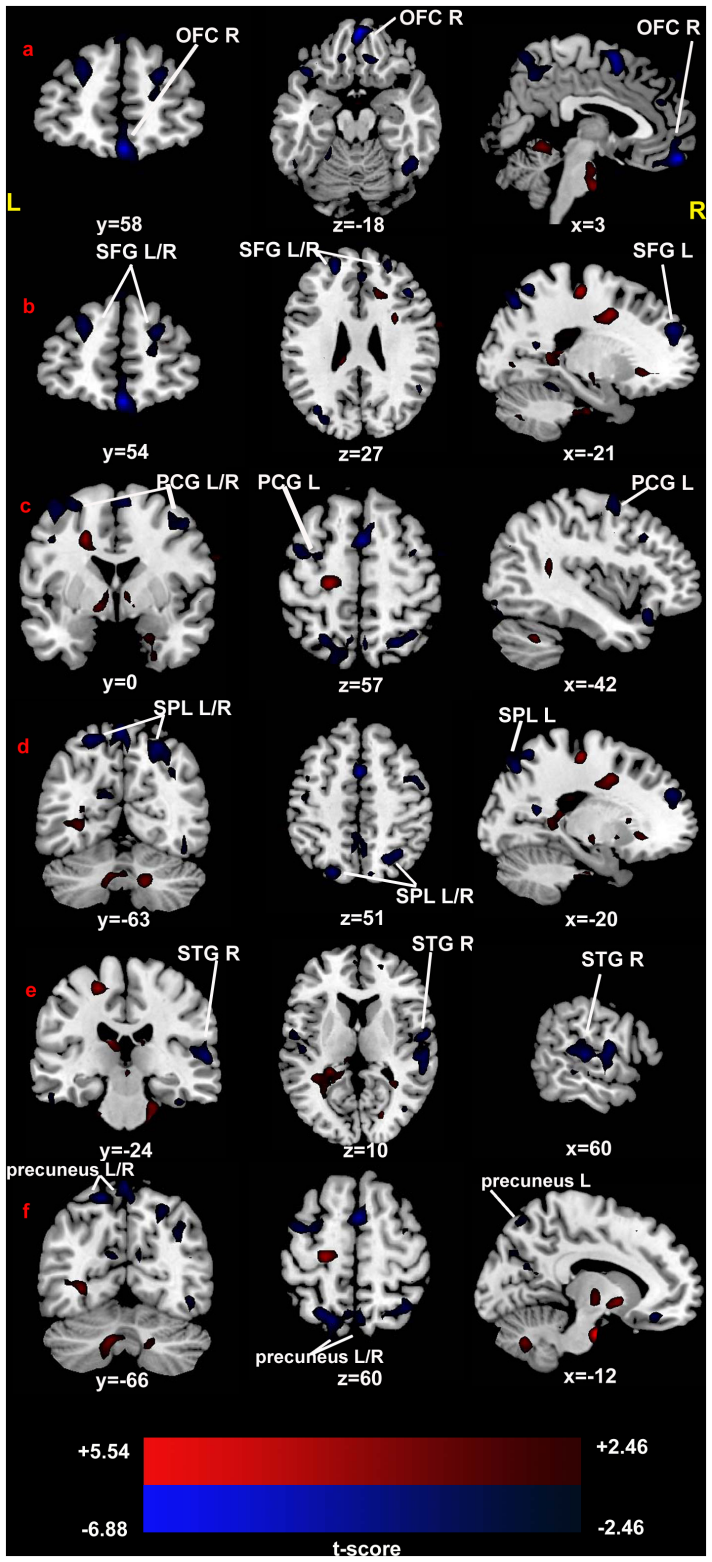
T-statistical different maps between the PBD patients and healthy controls (two-sample t test; P<0.05, corrected). Hot and cold colors indicate increased and decreased ReHo, respectively. T-score bars are shown at the bottom. The numbers beneath the images represent MNI coordinates. Abbreviation: OFC, orbitofrontal cortex; SFG, superior frontal gyrus; PCG, precentral gyrus; SPL, superior parietal lobe; STG, superior temporal gyrus; L, left; R, right.

**Figure 2:**
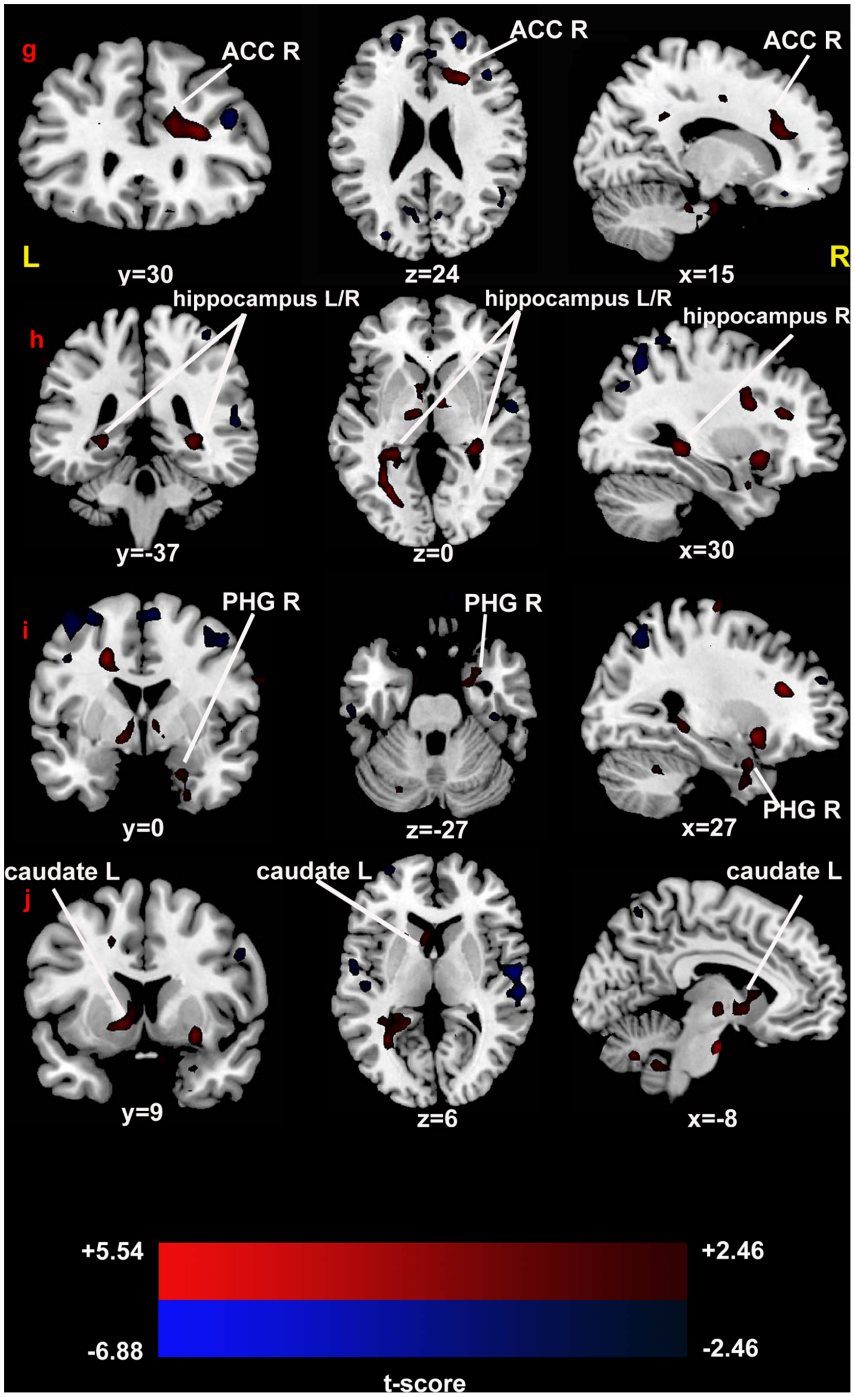
T-statistical different maps between the PBD patients and healthy controls (two-sample t test; P<0.05, corrected). Hot and cold colors indicate increased and decreased ReHo, respectively. T-score bars are shown at the bottom. The numbers beneath the images represent MNI coordinates. Abbreviation: ACC, anterior cingulate cortex; PHG, parahippocampal gyrus; L, left; R, right.

Compared with the healthy controls, the PBD patients showed significant increase of ReHo in the bilateral hippocampus, the right anterior cingulate cortex, the right parahippocampal gyrus, and the left caudate. Regions showed decreased ReHo included the bilateral precuneus, the bilateral precentral gyrus, the bilateral superior frontal gyrus, the bilateral superior parietal lobe, the right orbitofrontal cortex, and the right superior temporal gyrus. (Fig. 1; Fig. 2; Table 2).

**Table 2:** Significant Between-Group Differences in ReHo in PBD (n = 15) Versus HC (n = 15).

Brain region	Hem	BA	voxels	MNI Coordinates			T
				X	Y	Z	
Between-Group Analysis							
Decreased ReHo (PBD<HC)							
Precentral gyrus	L	6	43	−39	−3	60	−3.76
	R	6	20	45	3	48	−3.70
Orbitofrontal cortex	R	11	53	3	51	−9	−3.27
Superior frontal gyrus	L	10	24	−21	54	27	−3.99
	R	10	29	21	51	24	−4.21
Superior parietal lobe	L	7	90	−21	−75	51	−4.43
	R	7	80	24	−63	54	−4.22
Superior temporal gyrus	R	22	142	60	−24	6	−4.58
Precuneus	L	7	82	−12	−66	60	−2.87
	R	7	42	6	−57	48	−2.81
Increased ReHo (PBD>HC)							
Anterior cingulate cortex	R	32	30	15	30	24	3.59
Parahippocampal gyrus	R	28	28	27	3	−27	2.80
Hippocampus	L		26	−30	−36	−3	2.49
	R		30	30	−36	−3	3.84
Caudate	L		22	−9	12	6	2.84

Abbreviations: PBD, pediatric bipolar disorder participants; HC, healthy control subjects; BA, Brodmann Area; L,Left; R, Right.

### Correlations between ReHo and Manic Severity

**Figure 3:**
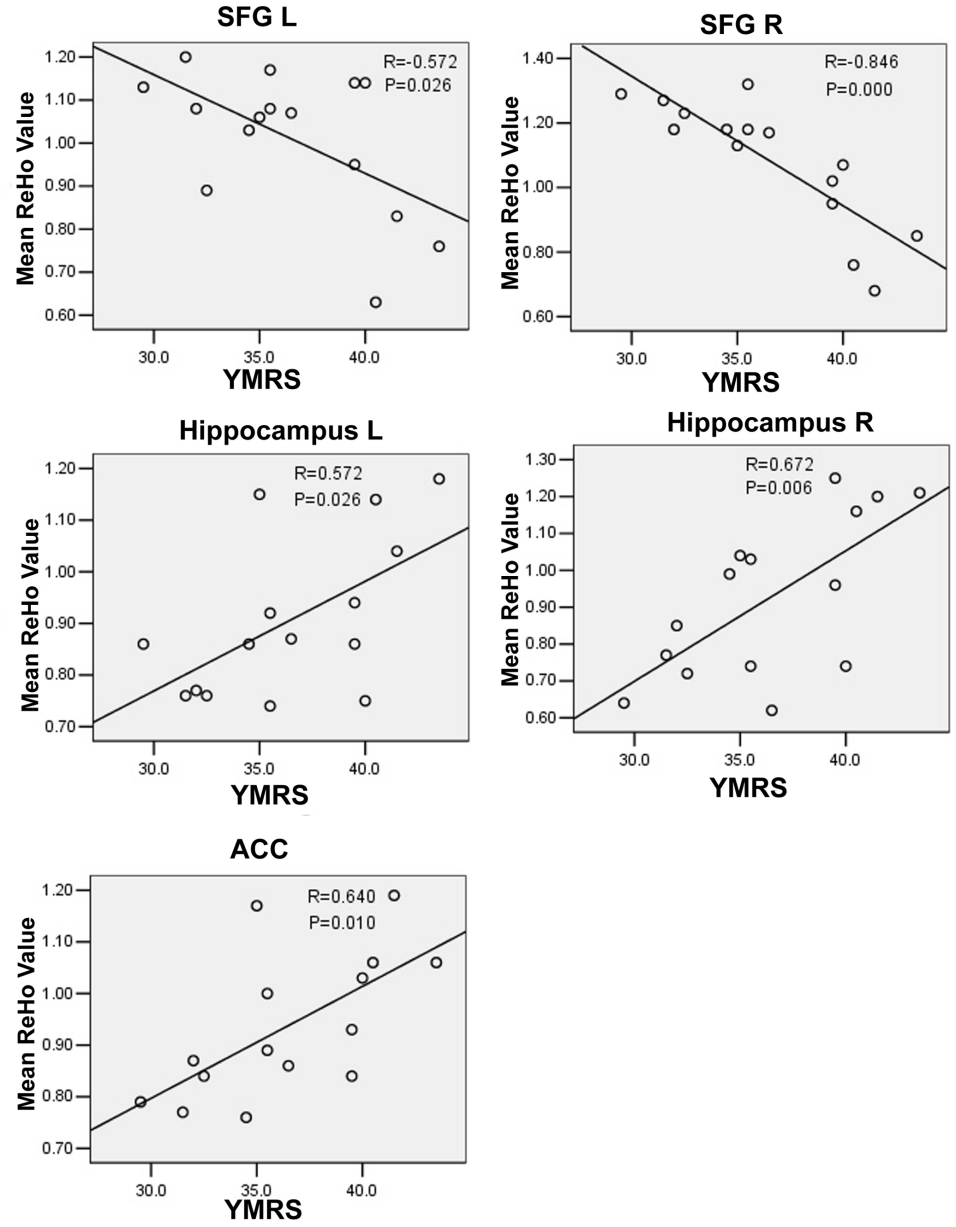
Correlation analysis between YMRS Score and ReHo in the PBD patients (P<0.05, corrected). Abbreviation: SPG, superior frontal gyrus; ACC, anterior cingulate cortex; L, left; R, right; YMRS, the Score of Young Mania Rating Scale.

Analysis by regressing ReHo at each ROI (regions of interest; the regions showed different ReHo in the above between-group ReHo analysis) against the score of YMRS in PBD group revealed significant positive correlation in the bilateral hippocampus (R = 0.572/0.672, P = 0.026/0.006) and the ACC (R = 0.640, P = 0.010). Negative relationship between ReHo and the Score of YMRS was found in the bilateral superior frontal gyrus in the PBD group (R = −0.572/−0.846, P = 0.026/0.000). (Fig. 3).

## Discussion

To our knowledge, this study is the first research that shows neural synchronization of local brain areas in PBD during resting state. The latest researches also showed that PBD was dysfunctional in ventral-affective and dorsal-cognitive circuits [4], [15], [41], but all these study used task-stimuli. Our altered ReHo during resting state might be the foundation of the activity change during task-state [42]. So from a whole new perspective, our results may present the baseline abnormality of the affective and cognitive circuits in PBD during resting state.

Studies of pediatric patients can provide important information about interactions between BD and brain development, and are less likely to be confounded by medication exposure, substance use, and the long-term effects of mood symptoms, compared with adult samples. Thus this study chose PBD as experimental subject.

Specifically, ReHo was developed to characterize the local synchronization of spontaneous fMRI BOLD signals and has the dominant characteristics of simple operation and good inter-experiment consistency [30]. Although ReHo method cannot directly reflect intensity of local neural activation, it can reflect neural synchronization of local brain areas. More specifically, increased ReHo may mean abnormal enhancement in emotional regulation [43]. On the contrary, reduced ReHo may present the loss of functional connectivity or dysfunction of coherence in the intraregional neural activity. In short, both increased and reduced ReHo may be related to abnormal brain activity [44].

### The Orbitofrontal Cortex (fig. 1a)

This result showed that the PBD manic patients had decreased ReHo in the orbitofrontal cortex (OFC). OFC is involved in the cognitive processing of decision-making [45]. Additionally, because of its functions in emotion and reward, the OFC is also considered to be a part of the limbic system [46]. When OFC connections are disrupted, varying cognitive, behavioral, and emotional problems may arise [47], [48]. This observation is supported by several previous studies on BD. Previous fMRI studies using cognitive tasks of response inhibition and decision-making have proposed increased or decreased OFC activation in BD [49], [50], [51]. However, using emotional face tasks, several studies showed increased or reduced activation in the OFC in BD and PBD [6], [52], [53]. From the above, both hypoactivity and hyperactivity of the OFC have been reported in BD in cognitive and affective tasks. Hypo- and hyperactivity in the OFC at the same time may reflect inhomogeneity and incoherence of OFC, which might explain reduced ReHo in the PBD.

### The Superior Frontal Gyrus (fig. 1b)

This study also found decreased ReHo in the superior frontal gyrus (SFG) in pediatric manic patients compared with healthy controls. Reduced ReHo in SFG represents decreased neural coherence in this region. The SFG was involved in cognitive process, such as executive function and memory retrieval [54]. Current studies found that the SFG was hyperactive in PBD [5] and the young first-relative of BD [46] during the working memory task. These data, along with our findings, suggest that the SFG is dysfunctional in PBD. Moreover, Lai et al. (2011) showed that patients with first-episode major depressive disorder displayed increased ReHo in the SFG after short-term duloxetine therapy, which suggests different neural mechanisms between BD and major depressive disorders or medication effects [55].

### The Precentral Gyrus (fig. 1c) and the Superior Parietal Lobe (fig. 1d)

When PBD patients are compared with HC patients, decreased coherences were also observed in the precentral gyrus and the superior parietal lobe (SPL), which are located in the cognition process network [56]. Furthermore,the precentral gyrus and the SPL have been identified in many cognitive studies such as inhibitory control task [57] and working memory (WM) task [58], [59] in PBD and BD. Taken together, these two brain regions constitute a fronto-parietal central executive network [60]. The findings of the current study reflect changes in the coherence of the precentral gyrus and the SPL.

### The Superior Temporal Gyrus (fig. 1e)

In this study, decreased ReHo was found in the superior temporal gyrus (STG). The STG takes part in the process of emotions [61]. Activity of the STG was either increased or reduced in remitted and symptomatic BD and PBD patients in emotional recognition and regulation [62], [63], [64]. Moreover, Dickstein et al.(2010)found negative functional connectivity between the DLPFC and the STG in PBD during resting state [29]. Functional connectivity is used to calculate temporal correlations between the time courses of remote brain regions. It may exhibit a long-distance interregional connectivity [65]. However, ReHo does not provide information about the synchronization among distant areas and just targets connectivity within the local region [39]. We speculate that the impaired functional connectivity between the DLPFC and the STG may due to inter-dysfunction in the STG, as found in the present study. Therefore, our findings do not contradict or copy previous functional connectivity studies. Instead, combining previous functional connectivity data and ReHo may increase our understanding of impaired prefrontal-temporal related network underlying the neurobiology of PBD.

### The Precuneus (fig. 1f)

In this study, we found decreased ReHo in the precuneus. It has been proposed that the precuneus is associated with episodic memory retrieval [66]. In recollection of memories, the precuneus could ‘judge’ whether this memory information was useful with the aid of the hippocampus [67]. The precuneus was found hyperactive in PBD patients during work memory [8]. Our finding also shows that precuneus is dysfunctional.

### The ACC (fig. 2g)

ReHo was significantly increased in the dorsal anterior cingulate cortex (dACC) in PBD patients, which reflects the enhancement of the local synchronization of spontaneous neural activities in this region. Increased ReHo may reflect neural hyperactivity in a regional brain area [30].

The dACC is the dorsal part of the ACC (BA32), which is connected with the parietal cortex, the prefrontal cortex as a central hub for processing top-down and bottom-top controls [68]. The dACC plays a part in many cognitive processes, such as attention processing [69], performance monitoring [70], response selection [71] and error detection [72]. Increased activity in the dACC has been reported in BD and PBD patients during the emotional go/nogo task [73], the working memory [8] and the inhibitory control [57] tasks. As mentioned before, ReHo increase observed in this region may support previous findings of dACC overactivity in PBD during cognitive regulation [8].

### The Hippocampus and the Parahippocampal Gyrus (fig. 2h,i)

ReHo of the hippocampus and the parahippocampal gyrus (PHG) were both increased in this study. Several studies exhibited increased hippocampal activation in BD and PBD patients in affective tasks involved in emotional faces [6], [74]. In fMRI studies, the PHG is always coactivated with the hippocampus using emotional tasks [75], [76]. The interaction of the two regions is intensively functional and anatomical, and is involved with emotional regulation [77]. BD patients exhibit emotional dysregulation involving in hippocampus and PHG [78]. Moreover, PBD patients also show increased activity in these two regions in both happy and angry faces [6]. Chen et al. (2010) performed a meta-analysis of different tasks used in fMRI studies of bipolar disorder and found that increased neural response exist in several regions, including hippocampus and PHG for emotional tasks [79]. As increased ReHo may reflect neural hyperactivity in a regional brain area [30], our results may add evidence to the previous findings.

### The Caudate (fig. 2j)

We also found ReHo increase in the caudate in the PBD patients compared to the healthy controls. The caudate is involved in both emotional and cognitive regulation [58], [73], [80]. Previous reports demonstrated increased activity in the caudate in BD patients implicated with emotional faces [80], working memory task [58], emotional go/nogo task [73], and a resting state [68]. PBD patients also showed increased activity in caudate during the task requiring inhibitory control and using positive stimuli [8], [81]. In the present study, we found increased ReHo in the caudate in the PBD group, which indicates that there is baseline brain activity impairment in PBD patients.

### ReHo and Manic Severity

We found positive correlation of abnormal ReHo in the hippocampus and the ACC with the manic severity (YMRS Score) in the PBD group. We also showed negative correlation between abnormal ReHo in the STG and the manic severity in the PBD group. The patients who had worse symptoms may have severer dysfunction in the neural activity. Combined with more large-sample experiments in the future, this finding will make it possible to assess clinical symptom severity in PBD patients.

### The Hypothesis of the Neural Circuits

The caudate was associated with the dorsal-cognitive circuits [73]. The dACC was involved in the dorsal-cognitive system [8]. The SFG and the SPL were also associated with the cognitive circuit, functioning in memory and attention [54], [56]. The OFC is a part of the VLPFC, which was a part of the ventral-affective circuit, according to the hypothesis of Pavuluri [31]. The hippocampus and the PHG were part of the limbic systems and might be involved with the affective circuit [77], [82]. The STG has taken part in the perception of emotions and also may be implicated with the affective circuit [61]. In summary, the SFG, the SPL, dACC and caudate are involved in dorsal-cognitive circuits; the OFC, the STG, the hippocampus and the PHG are implicated in ventral-affective circuits. Further studies are needed to elaborate this.

### Characteristics of Brain Regional Activity in Children and Adolescents

In this study, we found diffused activities in many brain regions, probably because that regional activity in children and adolescents were more diffused compared with adult [31]. Brain development in child is gradual. The prefrontal cortex attains an adult level of development, typically by the age of 15–16 years. The parietal cortex and the temporal cortex attain development by the age of 12 and 16 years, respectively. White matter has to be refined until 20 [31]. Children and adolescents are less influenced by psychological and social factors. Thus PBD may be more close to the nature of BD disease.

### Limitations

Firstly, almost all the PBD participants were taking psychotropic medications. It would be unethical to withhold anti-manic, anti-depressant and other antipsychotic medications for research purposes alone; and brief discontinuations would be ineffective, given their longer half-lives. In past fMRI studies of PBD, either no significant [80], [83] or ameliorative [84] effects in medication effects were reported. The medication seemed to normalize brain function of PBD patients. Therefore, our results were likely mainly from the disease rather than the medications, although we could not completely eliminate the medication effects.

Secondly, Although we excluded the prominent confounded factors of the comorbid ADHD and anxiety [85], we could not exclude all the comorbid diseases because of the characteristics of high comorbidity in PBD [3]. This study included two subjects with comorbid OCD and three subjects with psychosis. We can not do the post hoc analysis between these subgroups due to the quite small number. Moreover, it may less affect our results, because the number of patients with comorbidity is small.

Thirdly, although the PBD and control subjects were matched in age and gender, it need an larger study with sufficiently statistical power to meaningfully explore the potential confounding effects of age, gender and treatment in the future research.

### Conclusions

Our study for the first time shows neural synchronization of local brain areas in PBD during resting state. In summary, we find diffused dysfunctional regions associated with the ventral-affective and dorsal-cognitive circuits. The abnormal findings obtained from resting state-ReHo method are likely to be used for illustrating pathophysiologic mechanism of PBD. Combined with more large-sample experiments and clinical indicator in the future, this method may help in assistant diagnosis for PBD.
